# Phosphodiesterase type 4 expression and anti-proliferative effects in human pulmonary artery smooth muscle cells

**DOI:** 10.1186/1465-9921-7-9

**Published:** 2006-01-19

**Authors:** Ellena J Growcott, Karen G Spink, Xiaohui Ren, Saliha Afzal, Kathy H Banner, John Wharton

**Affiliations:** 1 Section on Experimental Medicine and Toxicology, Imperial College London, Hammersmith Campus, Du Cane Road, London W12 0NN, UK; 2 Pfizer Global Research and Development, Discovery Biology, Ramsgate Road, Sandwich, Kent CT13 9NJ, UK; 3 MRC London Neurodegenerative Diseases Brain Bank, Institute of Psychiatry, Windsor Walk, London SE5 8AF UK; 4 Novartis Institute for BioMedical Research, Wimblehurst Road, Horsham, West Sussex RH12 5AB, UK

## Abstract

**Background:**

Pulmonary arterial hypertension is a proliferative vascular disease, characterized by aberrant regulation of smooth muscle cell proliferation and apoptosis in distal pulmonary arteries. Prostacyclin (PGI_2_) analogues have anti-proliferative effects on distal human pulmonary artery smooth muscle cells (PASMCs), which are dependent on intracellular cAMP stimulation. We therefore sought to investigate the involvement of the main cAMP-specific enzymes, phosphodiesterase type 4 (PDE4), responsible for cAMP hydrolysis.

**Methods:**

Distal human PASMCs were derived from pulmonary arteries by explant culture (n = 14, passage 3–12). Responses to platelet-derived growth factor-BB (5–10 ng/ml), serum, PGI_2 _analogues (cicaprost, iloprost) and PDE4 inhibitors (roflumilast, rolipram, cilomilast) were determined by measuring cAMP phosphodiesterase activity, intracellular cAMP levels, DNA synthesis, apoptosis (as measured by DNA fragmentation and nuclear condensation) and matrix metalloproteinase-2 and -9 (MMP-2, MMP-9) production.

**Results:**

Expression of all four *PDE4A-D *genes was detected in PASMC isolates. PDE4 contributed to the main proportion (35.9 ± 2.3%, n = 5) of cAMP-specific hydrolytic activity demonstrated in PASMCs, compared to PDE3 (21.5 ± 2.5%), PDE2 (15.8 ± 3.4%) or PDE1 activity (14.5 ± 4.2%). Intracellular cAMP levels were increased by PGI_2 _analogues and further elevated in cells co-treated with roflumilast, rolipram and cilomilast. DNA synthesis was attenuated by 1 μM roflumilast (49 ± 6% inhibition), rolipram (37 ± 6%) and cilomilast (30 ± 4%) and, in the presence of 5 nM cicaprost, these compounds exhibited EC_50 _values of 4.4 (2.6–6.1) nM (Mean and 95% confidence interval), 59 (36–83) nM and 97 (66–130) nM respectively. Roflumilast attenuated cell proliferation and gelatinase (MMP-2 and MMP-9) production and promoted the anti-proliferative effects of PGI_2 _analogues. The cAMP activators iloprost and forskolin also induced apoptosis, whereas roflumilast had no significant effect.

**Conclusion:**

PDE4 enzymes are expressed in distal human PASMCs and the effects of cAMP-stimulating agents on DNA synthesis, proliferation and MMP production is dependent, at least in part, on PDE4 activity. PDE4 inhibition may provide greater control of cAMP-mediated anti-proliferative effects in human PASMCs and therefore could prove useful as an additional therapy for pulmonary arterial hypertension.

## Background

The survival of vascular smooth muscle cells is dependent on the balance between proliferation and apoptosis and the aberrant regulation of these pathways is implicated in proliferative vascular diseases such as pulmonary arterial hypertension (PAH); a progressive disease characterized by remodelling of distal pulmonary arteries [1]. Attention has therefore focused on therapies directed at suppressing proliferation and resistance to apoptosis in pulmonary artery smooth muscle cells (PASMCs) [2-4]. The ubiquitous second messenger cyclic adenosine monophosphate (cAMP) represents a potential target as it is one of the main intracellular factors regulating cell proliferation and apoptosis [5]. Prostacyclin analogues are an established vasodilator therapy for PAH that act mainly via IP receptors to stimulate adenylyl cyclase and intracellular cAMP levels [6], but also have anti-proliferative actions on human PASMCs, which may be important for their long-term effects *in vivo *[7,8]. The relationship between cAMP elevation and anti-proliferative potency of prostacyclin analogues is not necessarily clear [8], but additional strategies directed at elevating cAMP and amplifying the effects of prostacyclin signalling may be useful, particularly when the prostanoid is administered by repeated inhalation [9].

Phosphodiesterase (PDE) enzymes are responsible for the hydrolysis of the cyclic nucleotides and therefore have a critical role in regulating cAMP levels and downstream signalling in the cardiovascular system [10]. Eleven families of PDEs have been identified and of these PDE4 is the main cAMP specific PDE identified in the lung and vasculature [11,12]. PDE4 proteins are encoded by four genes (*PDE4A, PDE4B, PDE4C *and *PDE4D*), which produce numerous PDE4 variants [10,13] and studies on rat pulmonary arteries [14] and isolated PASMCs [15] suggest that these genes may be differentially expressed in the pulmonary vasculature. The presence of PDE4 has been investigated in homogenates of large human pulmonary arteries [16], but not in distal regions of the human pulmonary vasculature. Together with PDE3 enzymes the PDE4 family contributes to the regulation of pulmonary vascular tone, PDE4 inhibitors inducing relaxation of pulmonary artery preparations [14,16,17] and amplifying agonist-induced vasodilator responses [18,19]. On the other hand, the role of PDE4 in modulating vascular structure is unclear, studies to date indicating that when used alone PDE4 inhibitors are capable of suppressing the migration of isolated smooth muscle cells [20,21], but appear to be less effective at inhibiting vascular smooth muscle cell proliferation [15,22].

The mechanisms underlying remodelling of pulmonary arteries in PAH are multifactorial and include abnormalities in signalling by the TGF-beta superfamily, serotonin receptors and transporter, potassium channels, endothelial-derived factors and growth factors [23,24]. Proteolytic enzymes are also thought to be involved, including elastase and matrix metalloproteinases (MMP) such as the gelatinases MMP-2 and MMP-9, which degrade collagen and elastin, regulate extracellular matrix (ECM) deposition, and contribute to smooth muscle cell migration and proliferation [25,26]. Activation of these enzymes also leads to the production of the ECM protein tenascin-C, which acts as a survival factor, promoting proliferation and suppressing apoptosis in PASMCs [2]. An additional and potentially important role of MMP-2 is the regulation of vascular tone and structure, via the cleavage of vasoactive peptides [27]. In patients with PAH, MMP-2 and membrane type 1-MMP (MT1-MMP), a cell-surface activator of MMP-2, are co-localized in pulmonary vascular lesions [28] and isolated PASMCs exhibit increased gelatinase activity compared with controls [29]. Previous studies have suggested the involvement of the cAMP signalling pathway in regulating MMP-2 and MMP-9 production in a variety of human cell types [30,31]. cAMP-elevating agents have also been found to suppress MT1-MMP activity [32] and upregulate tissue inhibitors of MMPs [33], however, it is uncertain whether agents such as prostacyclin analogues and PDE inhibitors modulate gelatinase activity in human PASMCs.

We therefore sought to establish (1) the expression of *PDE4A-D *genes in human distal PASMCs; (2) the contribution of PDE4 to cAMP hydrolytic activity in these cells; and (3) the role of PDE4 in regulating cAMP levels, DNA synthesis, proliferation, apoptosis and gelatinase activity, using selective PDE4 inhibitors alone and in combination with prostacyclin analogues.

## Methods

### Isolation of PASMCs and culture

Lung tissues were obtained at lung transplantation (emphysema n = 8; pulmonary fibrosis n = 2; unused donor n = 1) and at lobectomy or pneumonectomy for bronchial carcinoma (n = 3), with informed consent and local approval from Hammersmith and Brompton-Harefield Hospitals ethics committees. Distal pulmonary artery smooth muscle cells (PASMCs) were isolated from micro-dissected segments of artery (<1 mm external diameter), as previously described [7]. Explants were placed in Dulbecco's modified Eagle medium (DMEM) containing 20% (v/v) foetal bovine serum (FBS) and 1% (v/v) antibiotic/antimycotic at 37°C, 5% CO_2_. Cells were maintained in DMEM containing 5–10% FBS and used at passages 3–12. For experiments confluent cells were made quiescent by incubation with serum-free media for 48 h and responses to platelet-derived growth factor (PDGF)-BB (5–10 ng/ml), prostacyclin analogues (cicaprost, iloprost) and PDE inhibitors were determined, as described below. When confluent, these cell isolates formed sheets of spindle-shaped cells and, like smooth muscle cells in the medial layer of intact distal human pulmonary arteries, expressed α-smooth muscle actin, calponin, endothelin ET_A _and ET_B _receptors and phosphodiesterase type 5 [7,34].

### PDE4 gene expression

Total cellular RNA was prepared using RNeasy Mini Kits and 0.2 μg of RNA was reverse transcribed into cDNA using a Superscript™ first strand synthesis kit (Invitrogen Ltd., Paisley, Scotland, UK). PCR was performed using proof start DNA polymerase (Qiagen Ltd., Crawley, West Sussex, UK), using 2 μl of reverse-transcribed cDNA solution (25 μl total volume), for 25–33 cycles with denaturation at 94°C for 30 sec, annealing at 55°C (β-actin) or 57°C (PDE4) for 30 sec and extension at 72°C for 1 min. This was followed by a final extension for 7 min at 72°C. After amplification, 10 μl of PCR product was separated using electrophoresis on a 1% (w/v) agarose gel and bands were identified using a ChemiGenius BioImaging system (Syngene, Cambridge, UK). The PDE4 primer sequences have been previously published [35] and PCR product identity was confirmed by cloning into *E.coli *using Zero Blunt Topo PCR cloning kit (Invitrogen) and sequencing (Lark Technologies Inc., Takeley, Essex, UK).

### Phosphodiesterase activity assay

cAMP-PDE activity was determined using a procedure modified from the Thompson and Appleman two-step conversion method, as previously described [34]. Briefly, cAMP-PDE activity was measured in both cytosolic and membrane fractions of PASMCs with 0.5 μM substrate (0.1 μM ^3^H-labelled cAMP, 0.4 μM unlabelled cAMP), and characterised using selective PDE inhibitors.

### Intracellular cAMP levels

cAMP levels were determined using an Adenylyl Cyclase Activation Flashplate^® ^assay (PerkinElmer Life and Analytical Sciences, Boston, MA), according to the manufacturers instructions. Briefly, cells from a T175 cm^2 ^cell culture flask were trypsinised, washed once in phosphate buffered saline (PBS) without calcium or magnesium and re-suspended in stimulation buffer without IBMX. Re-suspended cells (50 μl/well) were treated with PDE inhibitors for 10–20 min before the addition of prostacyclin analogues or forskolin for 60 min at 37°C. This time point was selected on the basis of our earlier observations, showing a maximal response to cicaprost in human PASMCs [7]. Detection buffer (100 μl), containing [^125^I]-cAMP, permeabilizer and 0.09% sodium azide, was added, incubated for 3 h at room temperature, and radioactivity counted using a TopCount NXT microplate counter (Packard, Pangbourne UK). Unlabelled cAMP standards (10–1000 pmol/well) were included in the same plate and results expressed as pmol cAMP produced per 10^5 ^cells, with at least 4 replicates per treatment.

### DNA synthesis, cell proliferation and apoptosis

DNA synthesis was measured by [^3^H-methyl]-thymidine incorporation over 24 h. Cells were seeded in 48-well plates (5 × 10^4 ^cells/well) in DMEM containing 5% FBS, allowed to adhere overnight, and then quiesced for 48 h in serum-free DMEM. Cells were subsequently incubated in fresh medium containing 0.25 μCi/well [^3^H-methyl]-thymidine, in the presence of PDGF-BB (5–10 ng/ml). PDE inhibitors and/or prostacyclin analogues were added 30–45 min before the addition of mitogen and [^3^H-methyl]-thymidine and the incorporation of thymidine was determined by liquid scintillation analysis, as previously described [7].

To determine cell proliferation, cells were seeded in 24-well plates (2 × 10^4 ^cells/ well) in DMEM containing 5% FBS and allowed to adhere overnight. The media was then replaced with fresh media containing drugs (4 replicate wells each) and changed every 2–3 days for up to 13 days. Adherent cells were trypsinised, counted and viability assessed by trypan blue exclusion.

The effects of PDE4 inhibition and cAMP signaling on apoptosis were assessed using Hoechst 33342 staining to define nuclear chromatin morphology and a cell death detection ELISA kit (Roche Diagnostics Ltd. (Lewes, Sussex, UK) to determine cytoplasmic histone-associated-DNA fragments. Cells were either maintained in media containing 5% FBS or serum-deprived for 48 h and treated with either iloprost (10^-10 ^to 10^-7 ^M) or roflumilast (10^-9 ^to 10^-6 ^M) for 48 h. PASMCs were cultured in 8-well permanox chamber slides (Lab-Tek™; Nalge Nunc International, Naperville, IL) for Hoechst 33342 staining (5 μg/ml for 20 min at 20°C) and individual nuclei were counted in at least 5 randomly selected fields for each well (>90 cells/field). The number of apoptotic cells exhibiting condensed nuclear fluorescence was determined and expressed as a proportion of the total cells. DNA fragmentation was determined using cells grown in 24-well plates, according to the manufacturers instructions.

### Gelatin zymography and matrix metalloproteinase production

Cells, seeded in 24-well plates (2 × 10^4 ^cells/well), were cultured in medium containing 10% FBS for at least 2 days before being serum-deprived for 24 h. Cells were incubated in fresh serum-free medium and stimulated with 10 ng/ml recombinant human tumour necrosis factor-α (TNF-α), interleukin-1β (IL-1β) or transforming growth factor-β1 (TGF-β1), phorbol 12-myristate 13-acetate (PMA, 10^-7 ^M) or the inactive phorbol ester 4α-PMA (10^-7 ^M), in the absence and presence of drugs at specified concentrations. The medium was collected after 48 h and gelatinase (MMP-2 and MMP-9) activity visualized by zymography and measured using MMP-2 and MMP-9 human Biotrack™ ELISA systems (Amersham Biosciences UK Ltd., Little Chalfont, Bucks, UK), according to the manufacturer's instructions. Conditioned medium was separated, under non-reducing conditions, in an 8% SDS-polyacrylamide gel, containing 1 mg/ml gelatin, at 4°C. After electrophoresis, gels were incubated in 2.5% Triton X-100 (twice for 15 min) to remove SDS, washed in water and incubated overnight at 37°C in buffer containing 50 mM Tris-HCl (pH 8.0), 5 mM CaCl_2_, 1 μM ZnCl_2 _and 0.1% Triton X-100. After fixation in 25% isopropanol and 10% acetic acid for 10 min, gels were stained in 0.25% Coomassie blue for 1–2 h. and destained in fixing/destain solution until bands of activity were clearly visible. The presence of MMP activity was confirmed by inhibition with 10 mM EDTA and the use of purified gelatinases (Merck Biosciences Ltd., Nottingham, UK) following activation with 1.5 mM p-aminophenyl mercuric acetate (APMA).

### Statistical analysis

Data were expressed as mean ± SEM or 95% confidence interval (95% CI) and analysed using GraphPad Prism version 4.0 (GraphPad software, San Diego, CA). Comparisons were made using one-way analysis of variance, with a Tukey post hoc test, or student's *t*-test, as appropriate. A value of P < 0.05 was taken to be significant.

## Results

### PDE4 gene expression and activity

**Figure 1:**
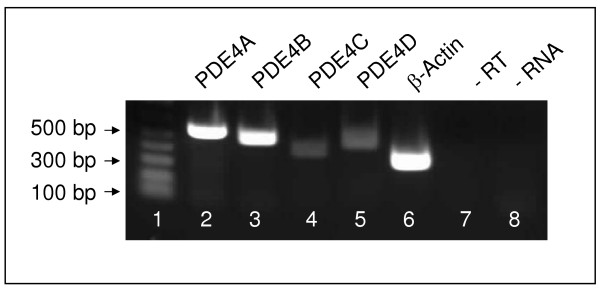
**Phosphodiesterase type 4 (PDE4) expression in human pulmonary artery smooth muscle cells**. RT-PCR demonstration of PDE4A (546 bp), PDE4B (506 bp), PDE4C (410 bp), PDE4D (479 bp) expression in a PASMC isolate, which is representative of 4 separate cell lines. Controls included expression of β-actin and absence of reverse transcriptase (- RT) or RNA (- RNA).

Products of 546, 506, 410 and 479 base pairs (bp), corresponding to fragments of PDE4A, PDE4B, PDE4C and PDE4D respectively were amplified by RT-PCR from distal human PASMCs total RNA (Figure 1). RNA amplification was not observed when either reverse transcriptase or RNA was omitted from the reaction, indicating that genomic DNA contamination was not present. The alignment of the sequenced RT-PCR products with corresponding regions in the human PDE4 isoforms confirmed their identity as PDE4 products (data not shown).

**Figure 2:**
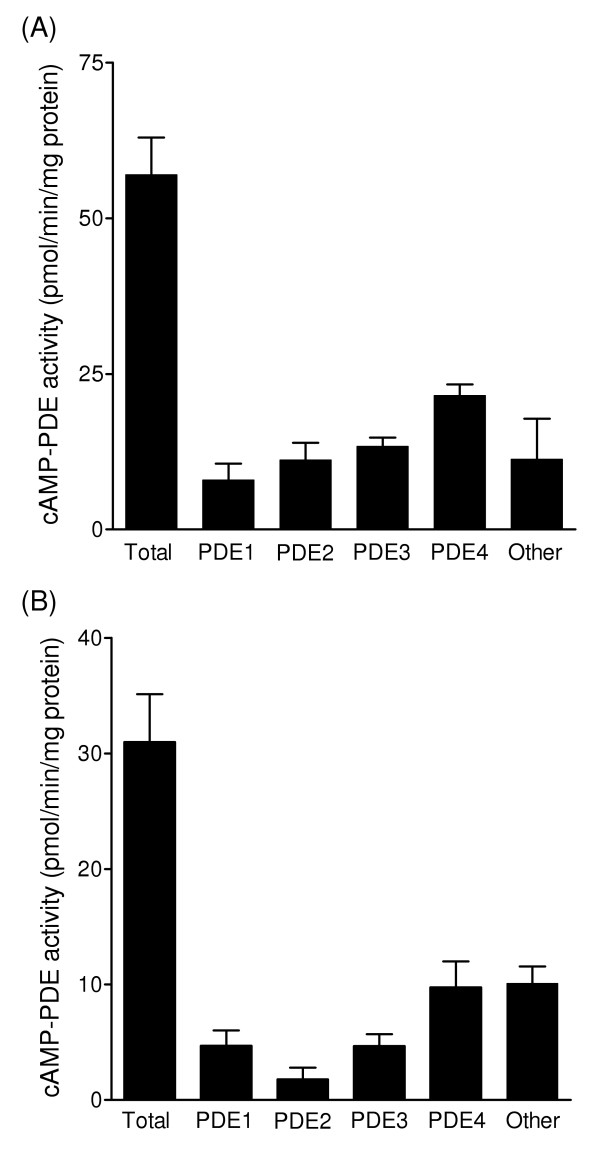
**Characterisation of cAMP phosphodiesterase (PDE) activity in human PASMCs**. Total cAMP hydrolytic activity and contribution of PDE enzyme families to cAMP hydrolysis in the cytosol (A) and membrane fractions (B) of human PASMCs (n = 5 isolates). Activity inhibited by 10^-3^} M EGTA (PDE1), 10^-5 ^M EHNA (PDE2), 10^-5 ^M cilostamide (PDE3) and 10^-6 ^M roflumilast (PDE4).

Both subcellular fractions displayed cAMP-PDE activity, the cytosol containing more activity than the membrane fraction (56.4 ± 6.4 versus 31.8 ± 3.9 pmol/min/mg protein; P < 0.001; n = 5 isolates). The hydrolytic activity was attenuated by the non-selective PDE inhibitor IBMX (5 × 10^-4 ^M), which reduced enzyme activity in both the cytosolic (100.7 ± 4.3% inhibition) and membrane fractions (78.1 ± 5.7% inhibition) respectively. PDE1 activity was determined by inhibition with 10^-3 ^M EGTA and the contribution of other enzymes using selective inhibitors of PDE2 (10^-5 ^M EHNA), PDE3 (10^-5 ^M cilostamide) and PDE4 activity (10^-6 ^M roflumilast). Each of these enzyme families contributed to the cytosolic and membrane cAMP-PDE activity (Figure 2A–B). PDE4 was the main specific cAMP hydrolytic activity demonstrated and contributed a greater proportion of the total activity (35.9 ± 2.3%; P < 0.01; n = 5) compared to PDE3 (21.5 ± 2.5%), PDE2 (15.8 ± 3.4%) or PDE1 activity (14.5 ± 4.2%).

### Effects of PDE inhibition on intracellular cAMP levels

**Figure 3:**
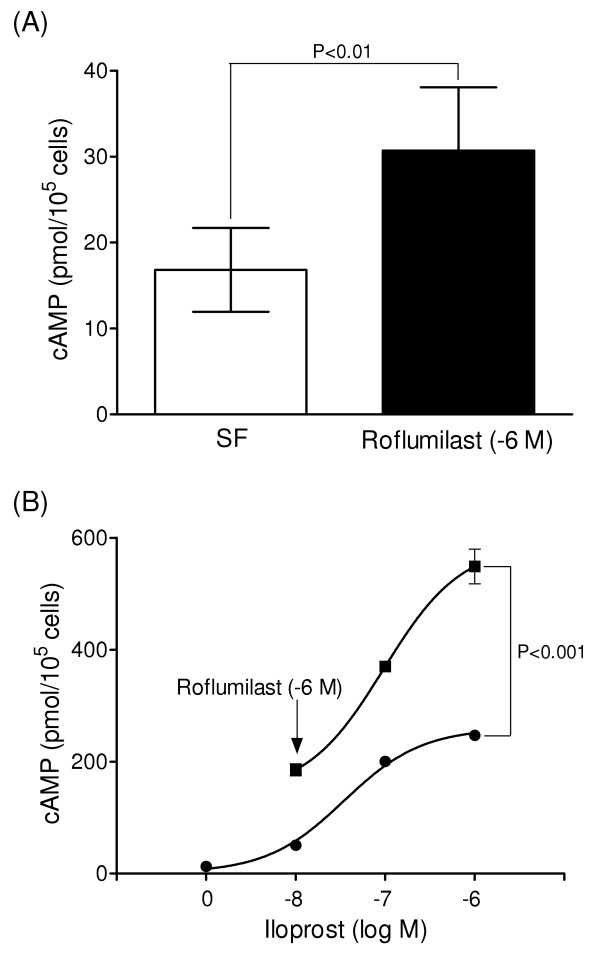
**Effect of roflumilast on intracellular cAMP levels**. Increase in cAMP levels following PDE4 inhibition with 10^-6 ^M roflumilast (A) and dual treatment with iloprost (B). Data (mean ± SEM) from 6 PASMC isolates (A) and four replicates, which is representative of three experiments with distinct isolates (B).

Treatment with roflumilast (10^-6 ^M) raised intracellular cAMP levels approximately 2-fold to 30.7 ± 7.4 pmol/10^5 ^cells (n = 6) (Figure 3A) and cilostamide (10^-6 ^M) induced a similar increase (39.2 ± 3.0 pmol/10^5 ^cells, n = 3). Stimulation of PASMCs with adenylyl cyclase activators, such as the prostacyclin analogue iloprost, had a concentration-dependent effect on intracellular cAMP levels, inducing a 4- (10^-8 ^M) to 19-fold (10^-6 ^M) elevation, which was augmented a further 2 to 3-fold by co-treatment with roflumilast (Figure 3B).

### Effects of PDE4 inhibition on DNA synthesis, cell proliferation and apoptosis

**Figure 4:**
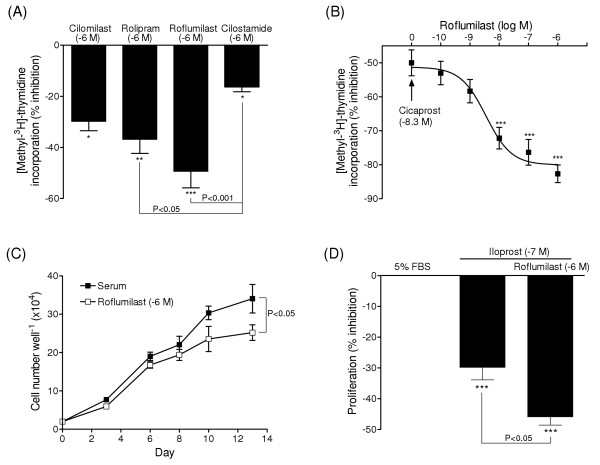
**Anti-mitogenic effects of PDE4 inhibition in PASMCs**. Effects of PDE4 (cilomilast, rolipram, roflumilast) and PDE3 (cilostamide) inhibitors on PDGF-BB (5 ng/ml) stimulated DNA synthesis (A). Concentration-dependent effect of roflumilast, combined with a sub-maximal concentration of cicaprost, on [methyl-^3^H]-thymidine incorporation (B). Effect of roflumilast (10^-6 ^M, open squares) on serum-stimulated (5% FBS) cell growth (closed squares) (C) and combined inhibitory effect of iloprost (10^-7 ^M) after 10 days serum-stimulated growth (D). *, P < 0.05; **, P < 0.01; ***, P < 0.001 versus control cells stimulated with either PDGF-BB (A), cicaprost (B) or serum (C-D). Data represent mean ± SEM from four-six distinct isolates (A-B, D) and four replicates (C).

Stimulation of PASMCs with PDGF-BB (10 ng/ml) increased [methyl-^3^H]-thymidine incorporation >4-fold over 24 hours (P < 0.001, n = 5). DNA synthesis was attenuated by both PDE4 (roflumilast, rolipram and cilomilast) and PDE3 (cilostamide) selective inhibitors, although the inhibitory effect of cilostamide was less than that observed following treatment with PDE4 inhibitors (Figure 4A). Co-treatment with cicaprost and PDE4 inhibitors also amplified the agonist-induced inhibition of DNA synthesis in a concentration-dependent manner (Figure 4B), with a rank order of roflumilast (EC_50 _value 4.4 nM; 95% CI [2.6 to 6.1 nM]; P < 0.001; n = 6), rolipram (EC_50 _value 59 nM; 95% CI [36 to 83 nM]; P < 0.01; n = 4) and cilomilast (EC_50 _value 97 nM; 95% CI [66 to 130 nM]; n = 4).

**Figure 5:**
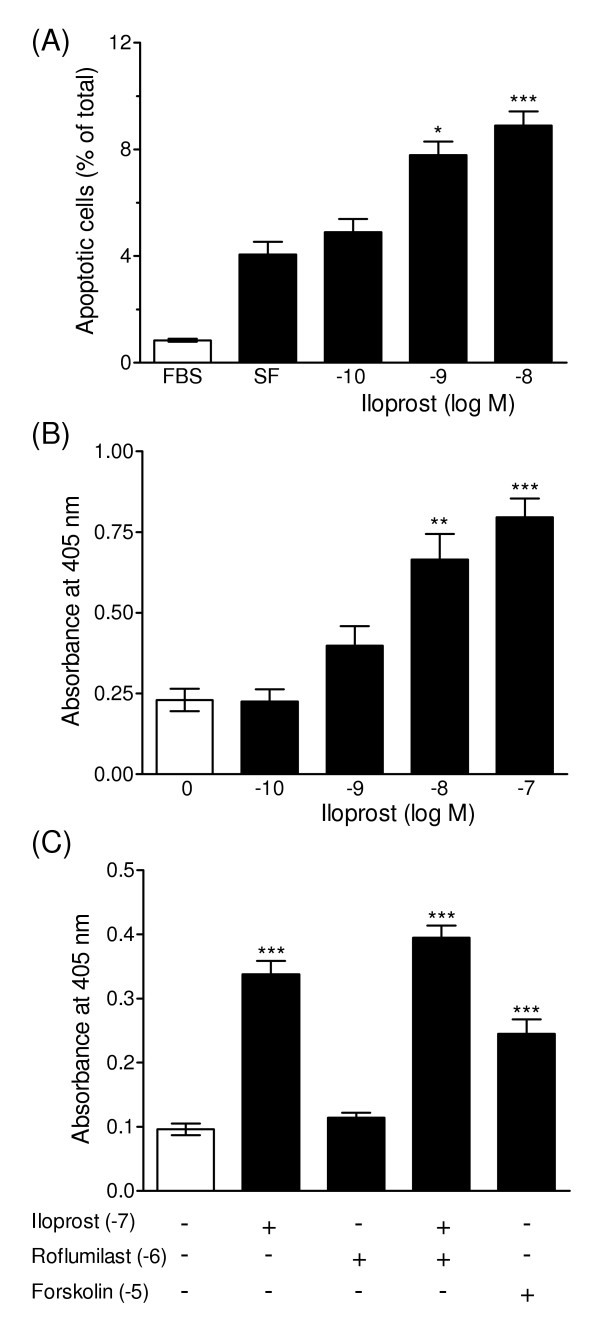
**Pro-apoptotic effects of cAMP elevating agents**. Concentration-dependent effect of iloprost on apoptosis, as demonstrated by the proportion of Hoechst-stained cells showing characteristic condensed nuclear fluorescence (A) and measurement of DNA fragmentation in human PASMCs (B). Effects of PDE4 inhibition (10^-6 ^M roflumilast) and adenylyl cyclase activation (10^-5 ^M forskolin) on DNA fragmentation (C). Data represent mean ± SEM of four replicates in three distinct isolates. * P < 0.05, ** P < 0.01 and *** P < 0.001 versus untreated control cells in serum free (SF) medium.

Roflumilast (10^-6 ^M) attenuated serum-stimulated PASMC proliferation (Figure 4C) as well as DNA synthesis and dual treatment with iloprost (10^-7 ^M) and roflumilast had a significantly greater anti-mitogenic effect (45.9 ± 2.7 % inhibition), compared to iloprost alone (29.8 ± 4.0 % inhibition; P < 0.05, n = 4 isolates) (Figure 4D). In addition to suppressing cell proliferation iloprost activated apoptosis, as demonstrated by a concentration-dependent increase in nuclear chromatin condensation and DNA fragmentation (Figure 5A–B). The adenylyl cyclase activator forskolin also induced an apoptotic response whereas treatment with PDE4 (roflumilast) and PDE3 inhibitors (cilostamide) alone had no significant effect on DNA fragmentation (Figure 5C). The combined effect of roflumilast and iloprost tended to be greater than iloprost alone, but overall the additional effect was not significant. The effects of PDE4 inhibition on DNA synthesis, cell proliferation and apoptosis were reproducible between different cell isolates, irrespective of whether they were derived from normal or diseased lung tissues.

### Effects of iloprost and roflumilast on MMP production

**Figure 6:**
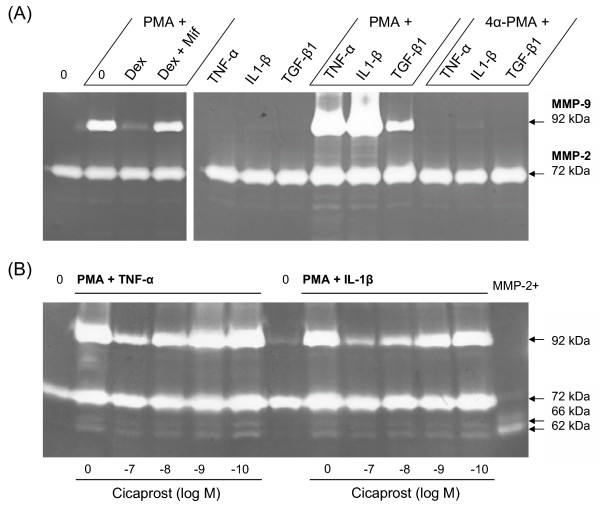
**Gelatin zymography of matrix-metalloproteinase (MMP) in conditioned medium from human PASMCs**. Representative zymograms showing the effects of phorbol 12-myristate 13-acetate (PMA, 10^-7 ^M) and 10 ng/ml TNF-α, IL-1β or TGF-β1 on inducible MMP-9 activity after 48 h (A) and the concentration-dependent inhibitory effect of cicaprost (B). MMP-9 (proMMP-9, 92 kDa); MMP-2 (proMMP-2, 72 kDa; active isoforms, 66 kDa and 62 kDa); MMP-2+, APMA-activated MMP-2; Dex, dexamethasone; Mif, mifepristone; 4α-PMA, inactive phorbol ester.

**Figure 7:**
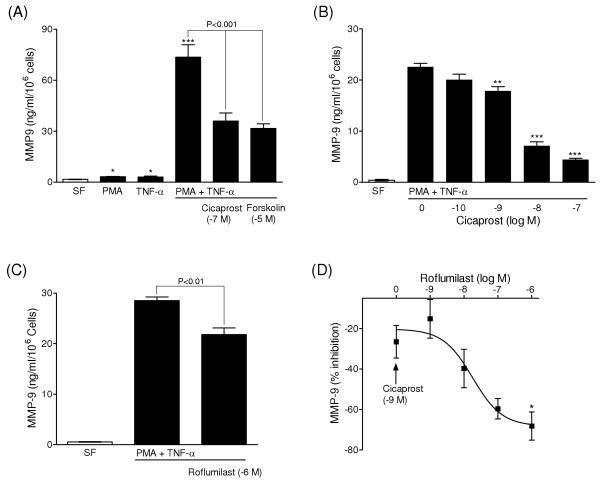
**Inhibitory effect of cAMP elevating agents on MMP-9 activity**. ELISA data of total MMP-9 activity in conditioned medium after 48 h, showing stimulation following treatment of PASMCs with PMA (10^-7 ^M) and TNF-α (10 ng/ml) and its inhibition by cicaprost and forskolin (A-B). Inhibitory effect of roflumilast, both alone (C) and in combination with a sub-maximal concentration of cicaprost (D). Data represent mean ± SEM of four replicates (A-B) and three-four distinct PASMC isolates (C-D). * P < 0.05, ** P < 0.01 and *** P < 0.001 versus medium from control cells in serum free (SF) medium (A) or PMA and TNF-α treatment (B-D).

**Figure 8:**
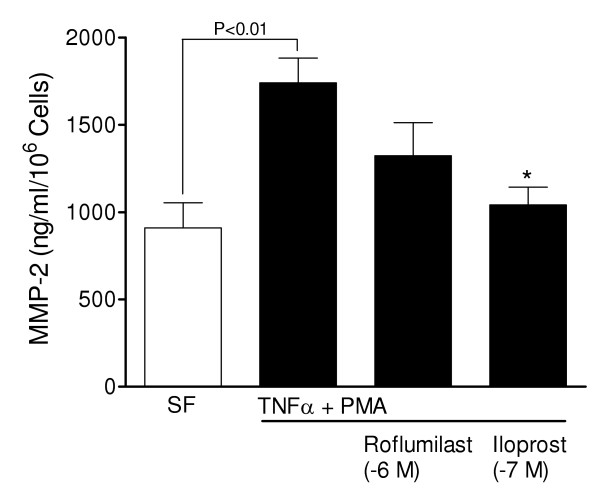
**Inhibitory effect of cAMP elevating agents on MMP-2 activity**. ELISA data showing the inhibitory effect of roflumilast (10^-6 ^M) and iloprost (10^-7 ^M) on PMA (10^-7 ^M) and TNF-α (10 ng/ml) stimulated MMP-2 activity in PASMC conditioned medium after 48 h. * P < 0.05, versus medium from control cells in serum free (SF) medium

Untreated, quiescent PASMCs displayed mainly pro-MMP-2 (72 kDa), rather than activated MMP-2 isoforms (66 kDa and 62 kDa), and did not display MMP-9 activity (Figure 6). Treatment of cells with PMA (10^-7 ^M) for 48 hours induced proMMP-9 (92 kDa), which was attenuated by dexamethasone and in turn blocked by co-treatment with the progesterone receptor antagonist mifepristone (Figure 6A). Stimulation with cytokines alone had relatively little effect on gelatinase activity, whereas dual treatment with PMA had a synergistic effect on MMP-9 induction (Figure 6A). The response was greater for TNF-α and IL1-β, compared to TGF- β1, and was not observed when the inactive phorbol ester 4α-PMA was used. MMP-9 activity was attenuated following stimulation with the adenylyl cyclase activator forskolin and, in a concentration dependent-manner, by the prostacyclin analogue cicaprost (Figures 6B &7A–B), suggesting regulation via the cAMP signalling pathway. Indeed, roflumilast attenuated MMP-9 activity and enhanced the inhibitory response to prostanoid stimulation in cells co-treated with a sub-maximal concentration of iloprost (Figure 7D). The treatment of cells with cytokines and PMA stimulated constitutive pro-MMP-2 (72 kDa) expression and activation of MMP-2 (66 kDa and 62 kDa) (Figures 6A–B), which was attenuated by roflumilast and iloprost (Figures 6 &8).

## Discussion

This study provides evidence indicating that the elevation of intracellular cAMP by prostacyclin analogues and PDE4 inhibitors suppresses proliferation and MMP activity and promotes apoptosis in distal human PASMCs.

The expression of *PDE4A, PDE4B, PDE4C *and *PDE4D *genes was detected in isolated human PASMCs. This is consistent with investigations demonstrating the expression of all four genes in systemic human arteries as well as other tissues [36] and contrasts with studies on rat pulmonary arteries [14] and isolated PASMCs [15] where PDE4 genes were found to be differentially expressed. In cultured human PASMCs, PDE3 and PDE4 represented the major cAMP hydrolyzing enzymes, the contribution of PDE4 being greater than that of PDE3. Studies examining PDE activity in extracts of human [16], bovine [17] and rat pulmonary arteries [37] have also demonstrated that PDE3 and PDE4 predominate, but with more PDE3 than PDE4 activity occurring in proximal regions of the pulmonary vascular bed, suggesting that there may be regional differences in the distribution of PDE4 activity. However, it should be borne in mind that the contribution of different PDEs to cAMP hydrolysis is a dynamic process, regulated by factors such as intracellular calcium levels and signalling activity. For example, we have shown that PDE1 activity is markedly induced in human PASMCs following stimulation with calcium and calmodulin [34] and it is now recognized that the cAMP-protein kinase A pathway regulates the expression [38] and catalytic activity of PDE4 variants [10,13] as well as the association of PDE4 enzymes with intracellular anchoring proteins [39].

Selective PDE4 inhibitors were found to attenuate DNA synthesis in human PASMCs. Of the inhibitors examined, roflumilast appeared most potent, the rank order of potency for the inhibition of DNA synthesis (roflumilast > rolipram > cilomilast) corresponding to that reported for the inhibition of human leukocyte cell functions [40] and inflammatory responses in experimental models of airway disease [41]. Roflumilast has also been identified as an oral anti-inflammatory treatment for chronic obstructive airway disease [42].

Cells treated with either a PDE4 or PDE3 inhibitor exhibited a comparable (~2-fold) increase in intracellular cAMP, whereas PDE4 inhibitors such as roflumilast were generally found to be more potent than cilostamide in suppressing PDGF-stimulated DNA synthesis. In previous studies on human airway smooth muscle cells and rat PASMCs, a similar disparity in the capacity of PDE inhibitors to elevate cAMP and modulate functions such as cell migration and proliferation was attributed to the intracellular compartmentalization of cAMP signalling [15,21]. Indeed, PDE4 isoforms are known to target particular intracellular sites and processes, resulting in the local regulation of cAMP generation and signalling, so that cAMP gradients within cells are likely to be more functionally relevant than cAMP levels in cells as a whole [13,39]. Nonetheless, prostacyclin analogues exhibited a greater capacity to elevate cAMP and inhibit DNA synthesis, proliferation and MMP production in PASMCs, compared to PDE4 inhibitors. Furthermore, in cells treated with submaximal concentrations of prostacyclin analogues, the combination of roflumilast and iloprost or cicaprost had a synergistic effect on cell function as well as cAMP levels. Thus, selective PDE4 inhibition may provide greater control of cAMP-mediated anti-proliferative effects in distal human PASMCs. Support for the therapeutic potential of combined treatment with a prostacyclin analogue and cAMP-PDE inhibitor comes from studies on monocrotaline- [43] and hypoxia-induced rat models of pulmonary hypertension [15], the anti-proliferative effects of iloprost *in vivo *being potentiated by the inhibition of PDE4 and/or PDE3 hydrolytic activity.

Such interaction is perhaps not surprising given the critical role of the cAMP-protein kinase A signalling pathway in regulating the expression, activity and intracellular localization of PDE4 isoforms in vascular smooth muscle cells [10,13,38,39]. However, PDE4 isoforms are widely expressed in mammalian tissues and PDE4 inhibitors, including cilomilast and roflumilast, have a low therapeutic ratio due to unwanted effects such as nausea and emesis [44]. Administering the PDE4 inhibitor by inhalation could overcome this limitation and because of the synergistic interaction between PDE4 inhibitors and prostacyclin analogues, it may be possible to achieve a greater therapeutic ratio by using a combination of these drugs. Indeed, selective PDE4 inhbitors for inhalation are in development (e.g. AWD 12–281) and a new generation of compounds is becoming available that appear to lack significant side effects (e.g. HT0712).

The induction of apoptosis in PASMCs may be beneficial in the remodelled pulmonary vasculature as novel therapies that reverse established pulmonary hypertension also induce apoptosis in these cells [2-4]. Importantly, both iloprost and forskolin induced apoptosis in human PASMCs, as demonstrated by nuclear condensation and DNA fragmentation. A similar apoptotic effect has been described in studies using isolated aortic smooth muscle cells [45], mediated via the cAMP-dependent inhibition of extracellular signal-regulated kinase (ERK) activity and stimulation of caspase-3 activity [46]. In these studies, stimulation of ERK activity suppressed apoptosis and because PDE4 isoforms are regulated by ERK[13] it was postulated that PDE4 activity was involved [46]. However, in the absence of a mitogenic stimulus, neither roflumilast nor cilostamide had an apparent effect on apoptosis in isolated human PASMCs.

We have demonstrated that the release of gelatinase activity from PASMCs is sensitive to cAMP elevating agents, including prostacyclin analogues and selective PDE4 inhibitors. The regulation of MMP-2 and MMP-9 release from human PASMCs may represent another mechanism contributing to the chronic effects of prostacyclin analogues in the hypertensive pulmonary vasculature. This contention is supported by reports of increased gelatinase activity in PASMCs from patients with PAH [29] and pulmonary vessels from rat models of pulmonary hypertension [25], and the finding that MMP-2 and MMP-9 is suppressed, together with vascular remodelling, in animals treated with iloprost and inhibitors of cAMP-PDE activity [43,47]. Furthermore, selective PDE4 inhibitors, but not PDE3 or PDE5 inhibitors, have been found to attenuate the release of MMP-2 and MMP-9, stimulated by PMA and cytokines such as TNF-α, from other human cells and tissues [48-51]. In agreement with studies on fibroblast cell lines [51,52], we also noted that dexamethasone attenuated the release of gelatinase activity from PASMCs and this may be significant in the light of recent findings indicating that prednisolone selectively inhibits the proliferation of PASMCs from patients with idiopathic PAH [53].

In conclusion, this study has demonstrated that PDE4 genes are expressed in human distal PASMCs. In addition to attenuating DNA synthesis and cell proliferation, stimulation of the cAMP signalling pathway was accompanied by increased apoptosis and reduced MMP production. The effect of cAMP-stimulating agents was dependent, at least in part, on PDE4 activity, supporting the hypothesis that PDE4 enzymes have a role in the regulation of DNA synthesis, cell proliferation and gelatinase activity in human PASMCs. PDE4 inhibition may therefore prove to be useful as an additional therapy for the treatment of proliferative pulmonary vascular disease.

## Competing interests

The author(s) declare that they have no competing interests.

## Authors' contributions

EJG: carried out the major part of the experiments, participated in the study design and drafted the manuscript

KS: participated in the study design and molecular biology experiments

XR: participated in the apoptosis experiments

SA: participated in the zymography experiments

KB: participated in the study design and discussion of data

JW: participated in the design and coordination of the project and writing of the manuscript
